# In Vitro Assessment of the Potential Effect of Vertical Peri‐Implant Soft Tissue Thickness on Nonsurgical and Surgical Implant Surface Decontamination Methods

**DOI:** 10.1111/clr.14415

**Published:** 2025-02-10

**Authors:** Annika Rahner, Peter Eickholz, Jan‐Frederik Gueth, Tuǧba Zahn, Iulia Dahmer, Hari Petsos

**Affiliations:** ^1^ Department of Prosthodontics Goethe University Frankfurt/Main, Center for Dentistry and Oral Medicine (Carolinum) Frankfurt am Main Germany; ^2^ Department of Periodontology Goethe University Frankfurt/Main, Center for Dentistry and Oral Medicine (Carolinum) Frankfurt am Main Germany; ^3^ Institute of Biostatistics and Mathematical Modeling Goethe University Frankfurt/Main Frankfurt am Main Germany; ^4^ Private Practice Soest Germany

**Keywords:** decontamination, dental air abrasion, dental implants, peri‐implantitis

## Abstract

**Objectives:**

In vitro analysis of the influence of vertical peri‐implant soft tissue thickness (STT) on nonsurgical and surgical implant surface decontamination efficacy.

**Material and Methods:**

A total of 360 implants were dipped in indelible color to imitate biofilm contamination, distributed to 30°, 60° or 90° angulated bone defect models and in subgroups of 40 assigned to a decontamination method (CUR: curette; SOSC: soundscaler; APA: air powder abrasion). Of these, 20 were subjected to a simulated STT of 1.5 or 3.0 mm, of which 10 were cleaned within a nonsurgical (NST) or surgical (ST) treatment. Uncleaned implant surface was determined by photographs. Surface changes were assessed using scanning electron micrographs (SEM).

**Results:**

The overall cleaning efficacy decreased significantly (APA > SOSC > CUR, *p* < 0.001). Cleaning efficacy failed to show a significant difference between both STTs (STT1.5: 63.68%, STT3.0: 63.26%; *p* = 0.877). Within respective STT, cleaning efficacy differed depending on the method and the approach (STT1.5: CUR: *p* = 0.169, SOSC: *p* = 0.004, APA: *p* < 0.001; STT3.0: CUR *p* < 0.001, SOSC: *p* < 0.001, APA: *p* < 0.001; STT1.5/NST, CUR: 82.34%, SOSC: 74.98%, APA: 93.60%; STT1.5/ST, CUR: 79.85%, SOSC: 65.37%, APA: 50.12%; STT3.0/NST, CUR: 83.19%, SOSC: 70.85%, APA: 92.31%; STT3.0/ST, CUR: 80.00%, SOSC: 64.61%, APA: 46.49%). Analysis of variance revealed significant associations of color remnants with the approach, the method used, and the defect angulation (*p* < 0.001). SEMs showed less surface damages after use of APA.

**Conclusions:**

In this In Vitro study no statistically significant influence of STT on the efficacy of surface decontamination could be detected. Treatment method, defect angle and approach were confirmed as predictors for the cleaning efficacy.

## 1 Introduction

The number of dental implants being placed is increasing due to their ease of placement, high survival rates, and success (Brennan et al. 2010; Jofre et al. 2013). Thus, clinicians have to deal with an increased number of complications associated with implants. Generally, those complications are categorized as mechanical, technical, and biological (Henry et al. 1996; Taylor et al. 2004). The classification of biological complications is relatively new in the context of peri‐implant diseases and conditions, being introduced in 2018 (Berglundh et al. 2018). The classification clarifies the relevance of peri‐implant mucositis and peri‐implantitis, which occur in 43% of implant cases over 9 years (Berglundh et al. 2018; Derks and Tomasi 2015).

Peri‐implantitis is defined as inflammation of the peri‐implant soft tissue combined with the loss of implant‐supporting bone (Berglundh et al. 2018; Heitz‐Mayfield 2008). Biofilm is the main factor that causes peri‐implantitis (Heitz‐Mayfield and Lang 2010). Poor oral hygiene, smoking, a history of periodontitis (Heitz‐Mayfield 2008), diabetes (Venza et al. 2010), a lack of attached keratinized mucosa (Afrashtehfar et al. 2023), and noncompliance with supportive implant therapy (Frisch et al. 2020; Lin et al. 2019) are risk indicators of peri‐implant mucositis and peri‐implantitis (Sanz et al. 2012; Tomasi and Derks 2012). Since this biofilm cannot be controlled with the sole use of systemic antibiotics or the local use of chlorhexidine, mechanical debridement is essential for treating peri‐implantitis (Carcuac et al. 2016; Heitz‐Mayfield and Mombelli 2014). However, there is no standard treatment protocol yet, and different decontamination approaches (e.g., curette, sonic scaler, air‐polishing, titanium brush, and laser) have been described (Heitz‐Mayfield and Mombelli 2014; Sahrmann et al. 2015; Schwarz et al. 2008).

Peri‐implantitis can be treated nonsurgically or surgically. While surgical treatment is more effective in terms of mechanical debridement, nonsurgical, and surgical treatment outcomes are unpredictable (Daubert and Weinstein 2019; Iatrou et al. 2021; Keim et al. 2019; Polak et al. 2017; Ronay et al. 2017; Sahrmann et al. 2015; Steiger‐Ronay et al. 2017; Suarez‐Lopez Del Amo et al. 2016; Tuchscheerer et al. 2021). Implant surface access and the chosen decontamination method have been subject of numerous In Vitro studies (Iatrou et al. 2021; Keim et al. 2019; Korello et al. 2023; Ronay et al. 2017; Sahrmann et al. 2015, 2013; Tuchscheerer et al. 2021).

There are situations where it is not possible to remove the suprastructure before surgical or nonsurgical peri‐implant treatment, which restricts implant surface accessibility and reduces the efficacy of surface decontamination (Korello et al. 2023). Moderate evidence suggests that the risk for peri‐implantitis is higher if the emergence angle is convex or over 30° (Atieh et al. 2023; Katafuchi et al. 2018; Lops et al. 2022; Majzoub et al. 2021; Yi et al. 2020). The buccal emergence angle also influences postsurgical mucosal recession (Wang et al. 2022). In most cases of implant‐supported single crowns on bone‐level implants, an attempt is made to conceal the transition from the titanium abutment to the ceramic restoration using submarginal placement. Consequently, the emergence profile and angle directly depend on the available vertical peri‐implant soft tissue thickness (STT) (Puisys et al. 2023).

Scanning electron micrographs (SEM) are a proven method to analyze changes of the dental implant surface in In Vitro studies (Hofmann et al. 2023; Iatrou et al. 2021; Keim et al. 2019; Korello et al. 2023; Ronay et al. 2017; Sahrmann et al. 2015; Tuchscheerer et al. 2021).

This study aimed to assess the extent to which the cleaning efficacy of different implant surface decontamination methods (curette, sonic scaler, and air powder abrasion with glycine powder), different defect configurations (30°, 60°, and 90°), and different access approaches (surgical and nonsurgical) depend on the STT (1.5 and 3.0 mm) around implant‐supported single crowns. Moreover, implant surface damage was analyzed using SEM. The cleaning efficacy differentiated according to STT was assumed as the primary outcome, all others as secondary outcomes. It was hypothesized that the three methods of implant surface decontamination, different defect configurations, and simulated approaches would demonstrate significantly different cleaning effects depending on STT.

## 2 Materials and Methods

The study was planned in accordance with the recommendations for standardized guidelines for reporting in vitro studies (Checklist for Reporting In vitro Studies, CRIS) (Krithikadatta et al. 2014). The study setup was based on the existing literature (Iatrou et al. 2021; Keim et al. 2019; Ronay et al. 2017; Sahrmann et al. 2015, 2013; Tuchscheerer et al. 2021) following the approach of Korello et al. (2023). However, this study differs from previous studies due to the use of two different mucosa masks simulating two different STTs.

### 2.1 Color Preparation, Acrylic Models, and Implant Fixtures

**FIGURE 1:**
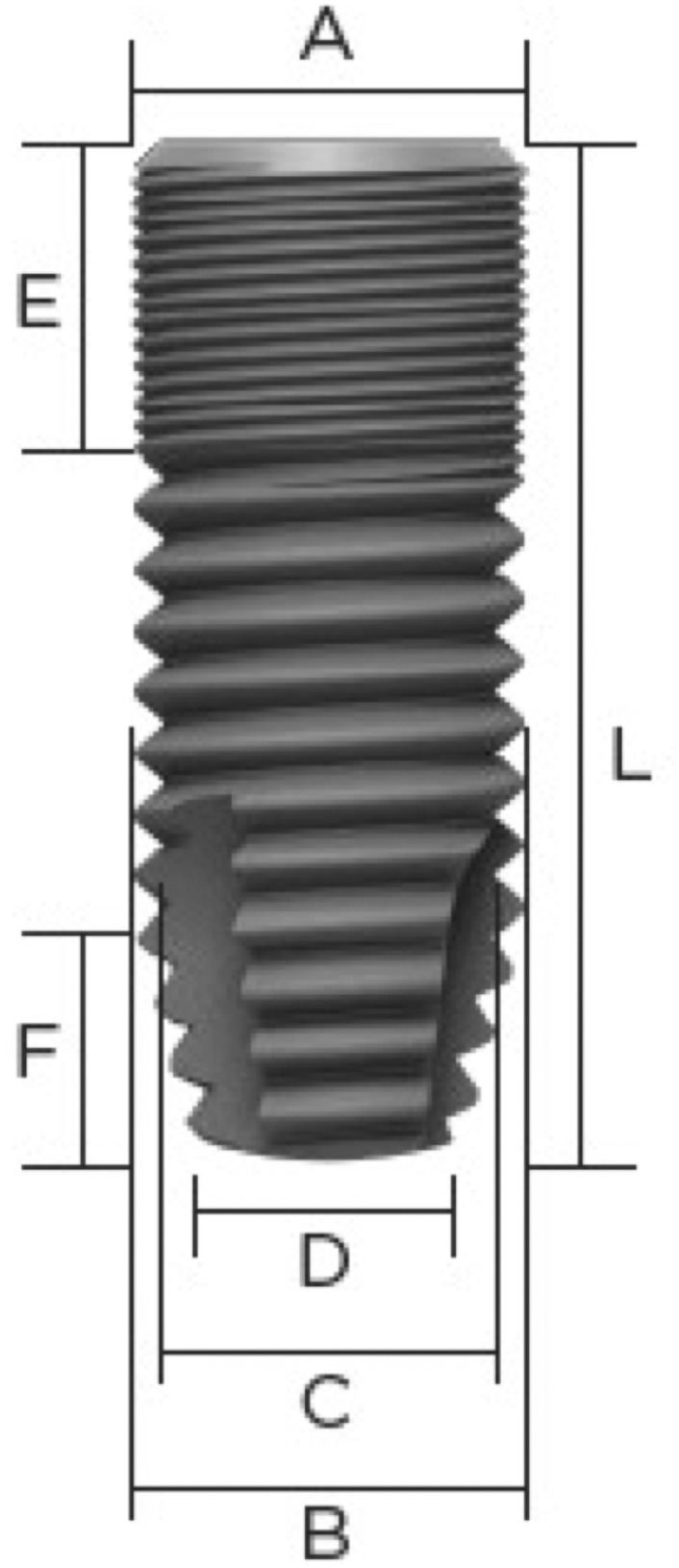
Technical drawing of the study implant (Astra Tech Implant EV 4.8 × 13S, Dentsply Sirona Implants, Mölndal, Sweden). The cervical diameter of the implant amounted 4.85 mm (A), the body core diameter 4.8 mm (B) rejuvenating in the apical 2.5 mm (F) from 4.2 mm (C) to a diameter of 3.7 mm (D) at the apex of the implant. The implant possessed a microthreated portion at the cervical 3.5 mm (E), with 0.22 mm pitch distance, by a total length of 13 mm (L) and a thread pitch distance of 0.66 mm at the implant body. Image courtesy of Dentsply Sirona.

For the biofilm simulation, 360 Astra Tech Implant EV 4.8 × 13S bone‐level implants (Dentsply Sirona Implants, Mölndal, Sweden) were completely dipped for 5 s into a permanent red color (Staedtler Lumocolor whiteboard, Nuremberg, Germany) using the Implant Driver EV Long (Dentsply Sirona, Hanau, Germany). The implant surface was not touched during this process. The implants were air‐dried shoulder‐down for 24 h. Concerning the implant specification, the technical information is shown in Figure 1. The implant surface roughness was 1.5 μm and was produced by blasting the surface with titanium dioxide particles and treating it with fluoride ions (Iatrou et al. 2021; Thor et al. 2007).

**FIGURE 2:**
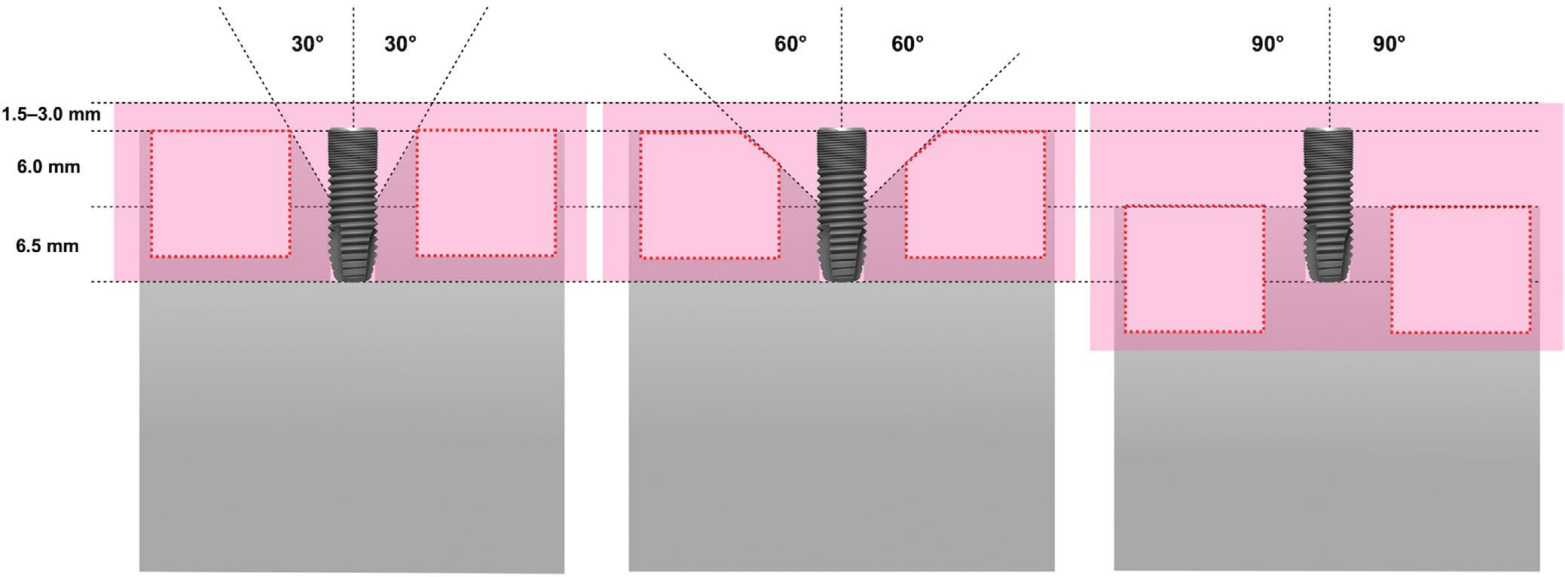
Drawings of the three different defect models 30° (intraosseous), 60° (intraosseous), 90° (supraosseous) with mucosal mask each with an exemplary inserted schematic draft of an untreated Astra Tech Implant EV 4.8 × 13S (Dentsply Sirona Implants, Mölndal, Sweden) implant. A peri‐implant soft tissue thickness of at least 1.5 or 3.0 mm was simulated in the nonsurgical approach. Dashed red line: Space for polymerization of the neighboring teeth.

**FIGURE 3:**
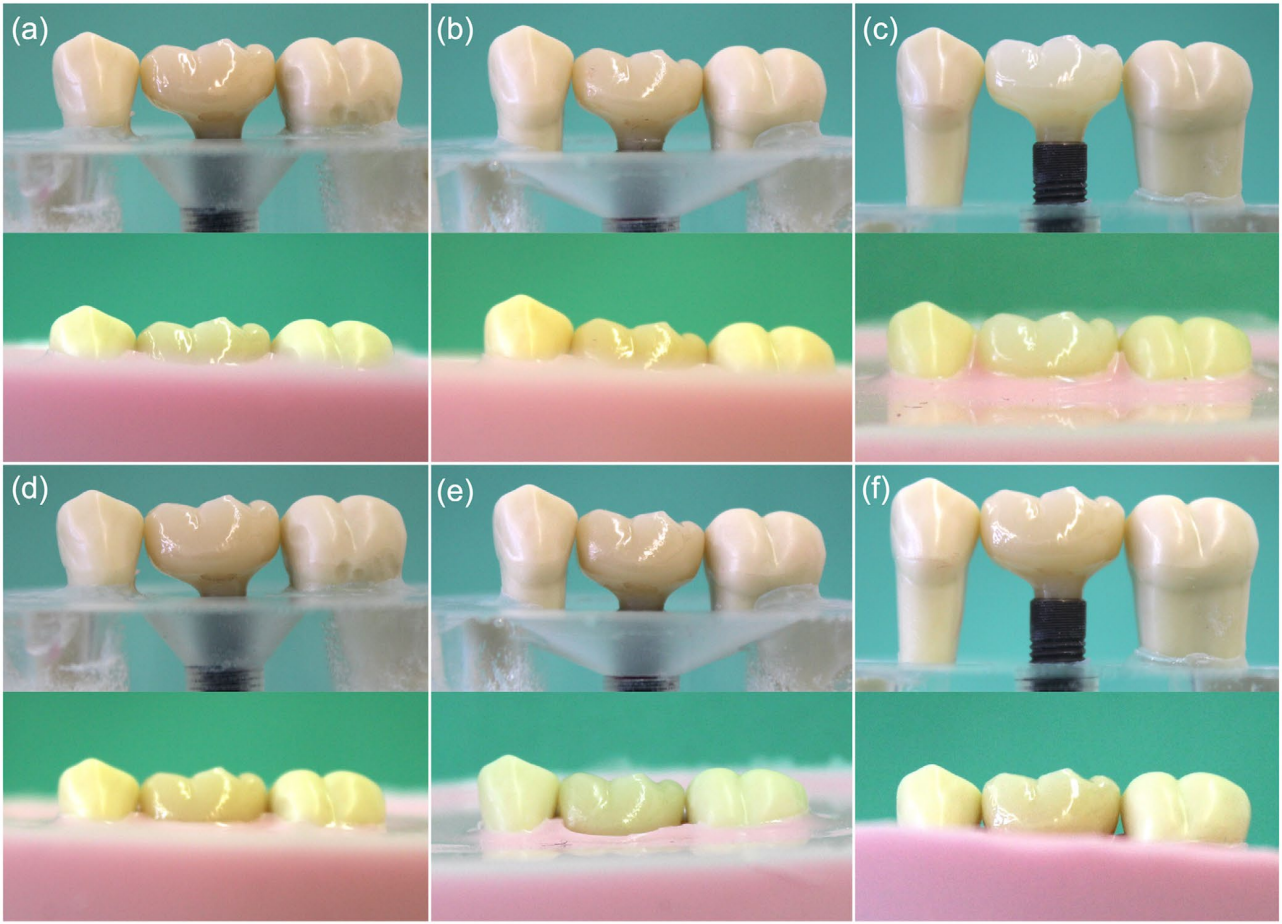
Lateral view of the three different defect angulations (a, d) 30°, (b, e) 60°, (c, f) 90° with implant‐supported crowns adapted to a peri‐implant soft tissue thickness of 1.5 mm (d–f) or 3.0 mm (a–c).

The in vitro models were created using computer‐aided design and manufactured out of acrylic glass, with three different defect angulations (30° and 60° for intraosseous defects and 90° for supraosseous defects). These represented clinical defect classes Ic (30° and 60°) and II (90°) (Schwarz et al. 2007). The implants were screwed into the acrylic models so that a 6 mm coronal section of the rough implant surface was free of acrylic glass (Figure 2). Adjacent plastic teeth (ANA‐4, Frasaco GmbH, Tettnang, Germany) were polymerized into the acrylic model using a positioning template to simulate neighboring teeth in proximal contact (Figure 3).

### 2.2 Prosthetic Suprastructures and Mucosa Masks

**FIGURE 4:**
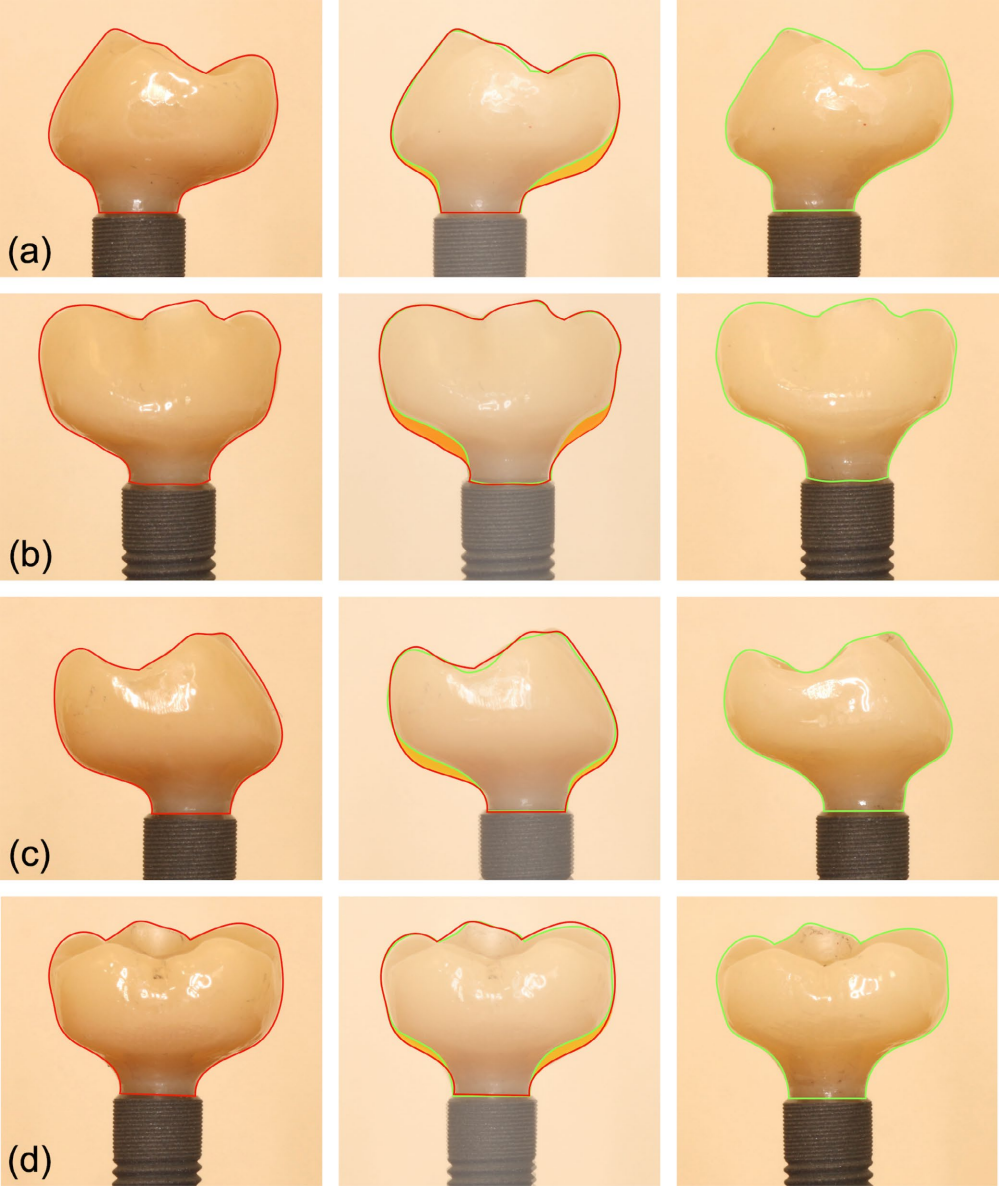
Mesial (a), buccal (b), distal (c), and lingual (d) view of the crown used adapted to a 1.5 mm peri‐implant soft tissue thickness (STT, left column) and 3.0 mm STT (right column). In the middle column, both crowns were roughly superimposed (STT1.5: Red outline, STT3.0: Green outline) and the difference in volume in the area of the emergence profile is marked in orange.

For each of the three defect models, two nonindexed, screw‐fixed, single multilayer zirconia crowns (DD cubeX^2^ ML [5Y‐TZP], Spenge, Germany) were manufactured with different concave emergence profiles corresponding to the two STTs (1.5 and 3.0 mm) (Figure S1; Figure 4). Consequently, in the experimental model each STT includes an associated suprastructure. To simulate the nonsurgical approach, the acrylic models were covered with individually manufactured, nontransparent silicone masks (Adisil rosé 1:1, SILADENT, Goslar, Germany) with a height of 1.5 or 3.0 mm manufacturing related with a possible height deviation of 0.25 mm. The 1.5 and 3.0 mm silicone masks were produced in an individual prefabricated hollow mold. The height of 1.5 and 3.0 mm were marked cervical around the crown and Adisil rosé was molded in until that mark. After hardening, the height of the silicone mask was measured at the inner circumference with the depth gauge of a digital caliper (Digital Vernier Caliper 150 mm, Hogetex, Varsseveld, the Netherlands). Thereby a precision of ±0.25 mm was detected at the inner circumference of the respective silicone mask. Further models were left uncovered to simulate the surgical approach (Figure 3).

### 2.3 Decontamination Protocols

After staining each of the 360 implants was treated using one of the following decontamination methods:

1. Group CUR: Langer steel curette SL5/6MF69E2 (curette: CUR [Hu‐Friedy, Chicago, IL, USA]).
2. Group SOSC: KaVo SONICflex 2003/L Airscaler (soundscaler: SOSC [KaVo Dental GmbH, Biberach, Germany]) with a steel tip (No. 60, KaVo) using level 2 power output adjustment with maximal activated irrigation.
3. Group APA: AIR‐FLOW Handy 2+ (E.M.S., Electro Medical Systems S.A., Nyon, Switzerland) with a PERIO‐FLOW handpiece (air powder abrasion: APA [E.M.S.]) using glycine powder (3 M Clinpro Glycine Prophy Powder, 3 M Germany GmbH, Neuss, Germany) and an attached PERIO‐FLOW nozzle tip (E.M.S.). A new nozzle tip was used for each implant, and the powder was refilled until maximum mark.

The basic settings were measured three times in advance for APA and SOSC, including drive air pressure (bar), water ejection (mL/min) (Korello et al. 2023), and, for APA only, powder emission rate (g/min) (Petersilka et al. 2002) (Table 1). Each implant was treated by one examiner (A.R.) for 2 min. The working distance and operation angle were chosen freely by the examiner. After each debridement cycle, the mucosa mask (for nonsurgical approaches) and the corresponding suprastructure were removed to enable the cleaned implant to be unscrewed. The detached color remnants were removed using a gentle 10 s air–water rinse (Sahrmann et al. 2015).

**TABLE 1:** Testing of the basic device settings.

Surface decontamination device	Water ejection (mL/min)				Powder emission rate (g/min)				Drive air pressure (bar)			
	I	II	III	Mean ± SD	I	II	III	Mean ± SD	I	II	III	Mean ± SD
**APA**	49.0	49.0	49.0	49.00 ± 0.00	4.7	3.5	3.5	3.90 ± 0.69	4.5	4.7	4.7	4.63 ± 0.12
**SOSC**	54.0	54.0	54.0	54.00 ± 0.00					3.5	3.5	3.5	3.50 ± 0.00

Abbreviations: APA, air powder abrasion; SD, standard deviation; SOSC, soundscaler.

### 2.4 Study Group Allocation and Inter‐Rater Reliability

**FIGURE 5:**
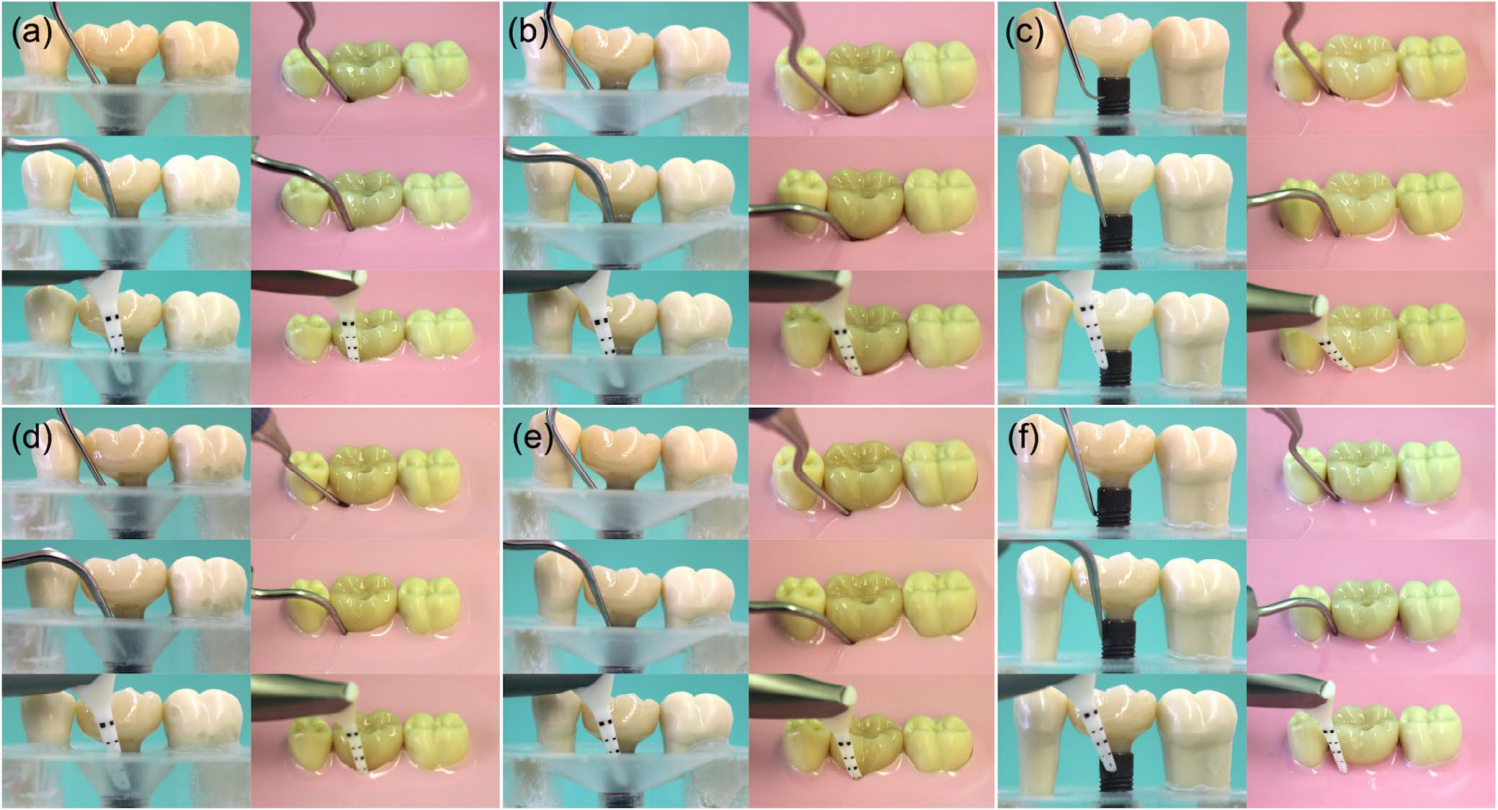
Oblique lateral view of the surgical and nonsurgical decontamination simulation in the three different defect angulations (a, d) 30°, (b, e) 60°, (c, f) 90° with implant‐supported crowns adapted to a peri‐implant soft tissue thickness of 1.5 (d–f) or 3.0 mm (a‐c) (1st and 4th column: Curette, 2nd and 5th column: Soundscaler, 3rd and 6th column: Air powder abrasion).

The 360 implants were distributed across three decontamination method groups, with 120 implants in each group (CUR, SOSC, and APA). 40 implants in each group were allocated to each defect angulation (30°, 60°, and 90°), which were then further subdivided into four groups, with 10 implants per group (Figure 5):

1. Peri‐implant soft tissue thickness 1.5 mm with corresponding suprastructure (1.5 mm STT) nonsurgical (NST).
2. Peri‐implant soft tissue thickness 3.0 mm with corresponding suprastructure (3.0 mm STT) nonsurgical (NST).
3. Peri‐implant soft tissue thickness 1.5 mm with corresponding suprastructure (1.5 mm STT) surgical (ST).
4. Peri‐implant soft tissue thickness 3.0 mm with corresponding suprastructure (3.0 mm STT) surgical (ST).

Group allocation and implant cleaning were performed using a randomization list (URL: www.random.org). A.R. was compared to an experienced examiner (H.P.) prior to the photo analyses to assess their level of agreement regarding photo assessment. For this, 10 treated implants (20 photos) were randomly selected and analyzed independently by both examiners. The aim was to achieve at least a high level of agreement (Cicchetti 1994).

### 2.5 Photo Documentation and Analysis

**FIGURE 6:**
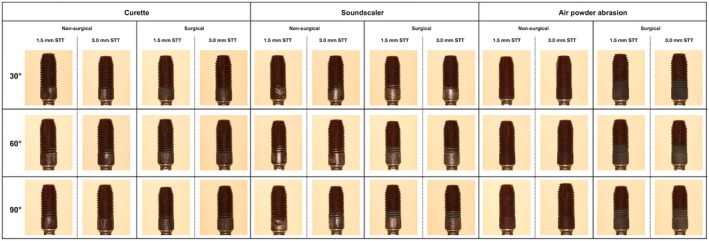
Exemplarily detailed images of cleaned implant surfaces according treatment method, to treatment approach, defect angle, and peri‐implant soft tissue thickness (STT).

Photo documentation was performed according to the procedures used in previous studies (Iatrou et al. 2021; Keim et al. 2019; Korello et al. 2023; Sahrmann et al. 2015; Tuchscheerer et al. 2021). All implants were placed using the Implant Driver EV Long (Dentsply Sirona, Hanau, Germany). The region of interest was not touched during this process. The implants were placed in individually manufactured nonmoveable holders in an equally illuminated photo tent (proxistar, Kastl, Germany). The examiner took digital photographs (Canon EOS 70D, Tokyo, Japan) of two sides of each implant (180° to each other) using standardized conditions and camera settings (distance 31.4 cm, ISO 100, aperture f/20, exposure time 1 s) and a ring flash (Canon MR‐14, Tokyo, Japan) (Figure 6).

The examiner analyzed the 720 photos captured using free image editing software (ImageJ 1.54f, US National Institutes of Health, Bethesda, Maryland, https://imagej.nih.gov/ij/). For each side, a constant region of interest (ROI) was selected by H.P. from the reference implant and saved as a mask. The ROI (output in pixels) included only the 6 mm surface that was accessible to the cleaning device and not the 6.5 mm apical region enclosed in acrylic glass. The corresponding ROI was matched to the photographed side. Red color remnants included in the ROI were marked by the image editing software. Basic values (brightness, contrast, sharpness) remained constant throughout the analysis. The total pixel number of the marked color remnants was measured and transferred into a custom‐made Excel data sheet. The corresponding value (pixel) of the contralateral implant side was also assessed. The total percentage of residual stained surface was calculated by evaluating the mean percentages of both implant sides.

**FIGURE 7:**
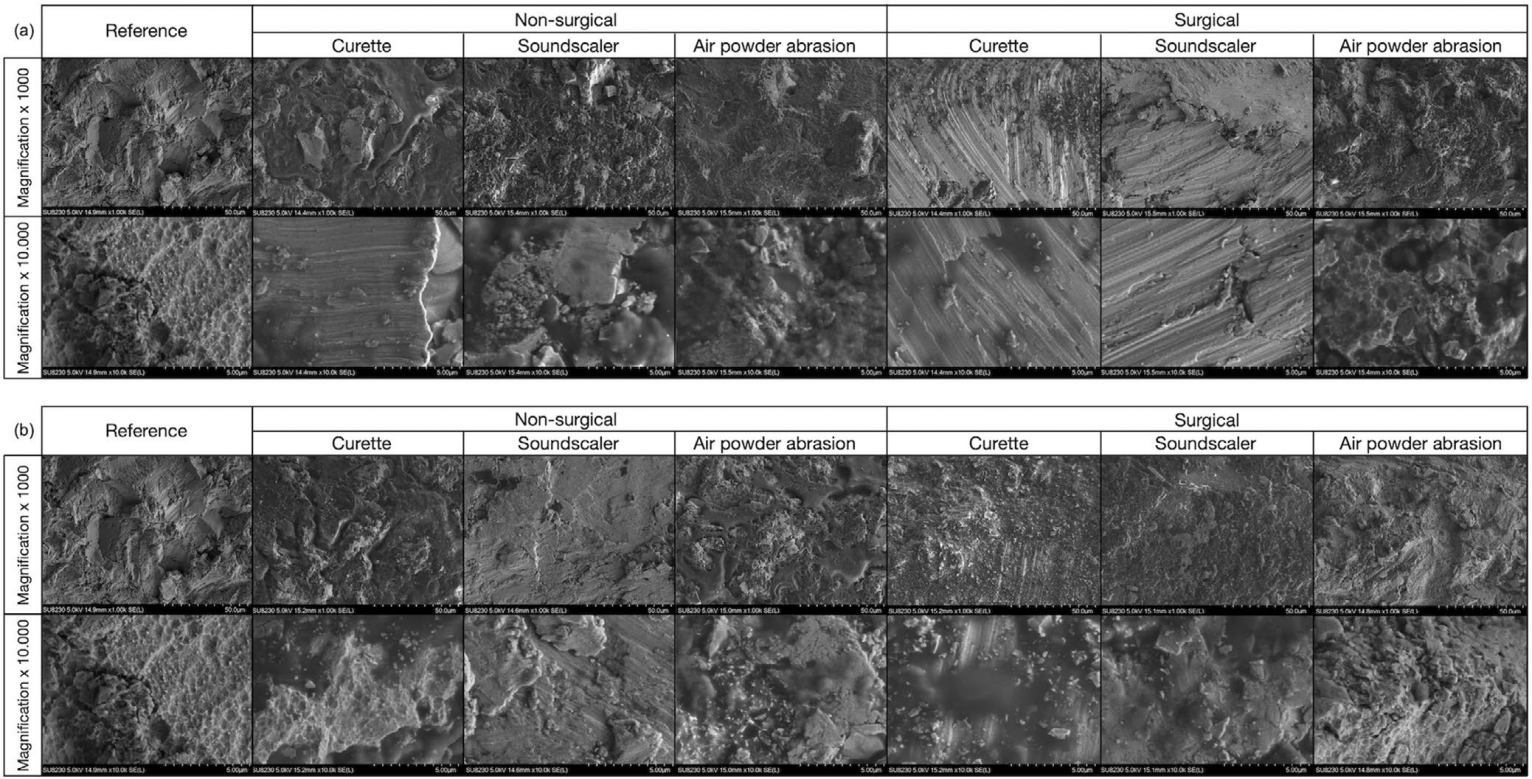
Scanning electron microscopy images of untreated (reference) and nonsurgically and surgically treated macro threads of the implant surfaces by different instruments within a peri‐implant soft tissue thickness of 1.5 mm (a) and 3.0 mm (b) at magnification of ×1000 and ×10.000.

Scanning electron microscope (SEM) images (SU8230 with a cold‐field‐emission cathode, 5kv, 15 mm distance, Hitachi, Tokyo, Japan) were taken of the color free area 1–2 mm apical the microthreated portion of the analyzed implant surface. One randomly selected surgical and nonsurgical treated implant across each treatment method and gingival height, in total 12 and the reference implant was examined using SEM (magnification 1 × 1000 and 1 × 10 000; Figure 7). The aim of this assessment was to evaluate the influence of the different decontamination protocols on the implant surface by visual examination of the SEM on scratches.

### 2.6 Statistical Analysis

The implant was considered the statistical unit. For each implant, the percentage of uncleaned surface was determined by calculating the mean value of the measurements from both sides of the implant.

To evaluate the interindividual calibration of the examiners (A.R., H.P.) for the measured percentages of remaining color remnants, interclass correlation coefficient (ICC) was calculated (< 0.4: poor agreement, 0.40–0.59: moderate agreement, 0.6–0.74: high agreement, ≥ 0.75: very high agreement) (Cicchetti 1994).

Cleaning efficacy across different treatment modalities, defect angulations, and/or STTs was analyzed using descriptive data (mean ± SD, median, lower/upper quartile, and interquartile range [IQR]). Because the data were non‐normally distributed based on the Kolmogorov–Smirnov test, intergroup comparisons were made using the Kruskal–Wallis test. To consider all factors that may have influenced the cleaning efficacy within the same model, analysis of variance (ANOVA) was performed. Significance level in all analyses was set at 5%. Statistical analysis was performed using the SPSS 26 software package (IBM Corp.) and R (packages “RVAIdeMemoire,” “PMCMRplus,” “rcompanion,” “stats,” “coin”).

## 3 Results

**FIGURE 8:**
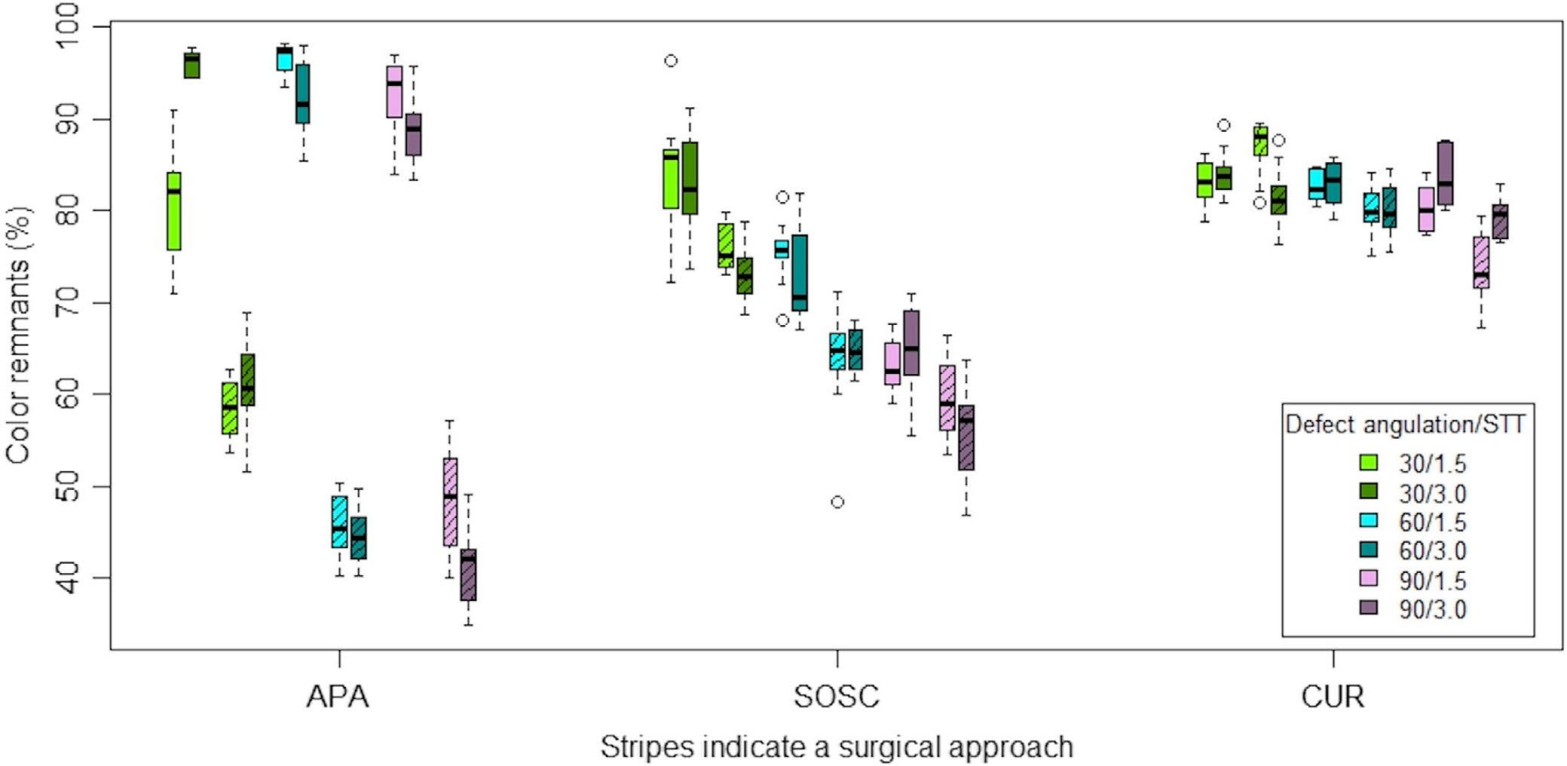
Box plot diagram showing the distribution of color remnants according to the used decontamination method, defect angle, approach, and soft tissue thickness (circles indicate outliers ≤ 3 interquartile ranges).

None of the studied implant surfaces were completely free (i.e., 0%) of color remnants. A trend in cleaning efficacy (CUR < SOSC < APA) is visible in the box plot diagram (Figure 8). A linear model was calculated with the four factors STT, approach, defect angle and method and their interactions. It was found that method, approach and defect angle were significant both globally (*p* < 0.001; *n* = 360) and in the interaction (*p* < 0.001; *n* = 360). STT was only significant in the interaction with approach (*p* = 0.033; *n* = 360). Both STT of 1.5 mm and STT of 3.0 mm showed significantly better cleaning results for the surgical than the nonsurgical approach (Table 2).

**TABLE 2:** Analysis of variance (ANOVA) for color remnants.

	Df	Sum of squares	Mean sum of squares	*F*	*p*
Predictors					
Approach	1	27482.1	27483.1	1125.25	< 0.001
Method	2	10366.8	5183.4	212.23	< 0.001
Defect angle	2	5749.8	2874.9	117.71	< 0.001
STT	1	1.3	1.3	0.05	0.815
Interactions					
Approach:STT	1	111.4	111.4	4.56	0.033
Method:STT	2	74.4	37.2	1.52	0.220
Defect angle:STT	2	116.6	58.3	2.39	0.093
Approach: method	2	26351.4	13175.7	539.45	< 0.001
Approach: defect angle	2	1152.2	576.1	23.59	< 0.001
Method: defect angle	2	2130.1	532.5	21.80	< 0.001
Residuals	4	8304.2	24.4		

*Note:* Dependent variable: color remnants (%).

Abbreviations: Df, degrees of freedom; STT, soft tissue thickness.

### 3.1 Inter‐Rater Reliability and Basic Device Setting Measurements

The examiners (A.R., H.P.) showed significant and positive high to very high level of agreement in their photo assessments (ICC = 0.914 (95% CI: 0.645, 0.979); *p* < 0.001; *n* = 10) (Cicchetti 1994). The SD of individual measurements was 0.06%. The results of the basic device setting measurements for APA and SOSC are shown in Table 1.

### 3.2 Soft Tissue Thickness

Cleaning efficacy failed to show a significant difference between the STTs (STT1.5: 63.68%, STT3.0: 63.26%; *p* = 0.877; *n* = 180). Therefore, the hypothesis cannot be confirmed. Within STT, APA achieved significantly better results with the ST approach compared to NST. The cleaning efficacy for the investigated methods across different approaches, STTs and defect angles varied inhomogeneously (Tables 3 and 4).

**TABLE 3:** Medians, means, and standard deviations (%) of residual colored surface areas after (a) nonsurgical and (b) surgical treatment with 1.5 mm peri‐implant soft tissue thickness with three different surface decontamination methods (CUR, SOSC, APA). Separately presentation for different treatment methods and defect angulations. *N* = 10 for each treatment method in each defect angulation.

Defect angulation	30°					60°					90°				
			Percentile (%)					Percentile (%)					Percentile (%)		
	Min. (%)	Max. (%)	25th	50th (Median)	75th	Min. (%)	Max. (%)	25th	50th (Median)	75th	Min. (%)	Max. (%)	25th	50th (Median)	75th
(a) *Nonsurgical*															
CUR	71.20	78.30	72.81	75.28	77.08	67.83	80.60	70.19	74.36	78.29	65.37	79.40	71.50	74.88	76.65
Mean ± SD (%)	75.09 ± 2.43					74.27 ± 4.20					73.82 ± 4.19				
SOSC	63.86	84.33	68.50	73.33	80.08	57.33	69.36	58.93	60.97	65.52	46.65	55.50	47.25	50.40	54.74
Mean ± SD (%)	74.04 ± 6.75					62.09 ± 3.91					63.06 ± 2.66				
APA	62.88	93.44	64.10	68.42	79.13	90.31	97.48	94.11	95.98	97.25	83.11	96.25	87.73	90.18	93.60
Mean ± SD (%)	72.82 ± 10.21					95.38 ± 2.31					90.29 ± 4.06				
(b) *Surgical*															
CUR	74.33	85.64	76.94	81.38	84.20	65.44	78.01	70.08	73.32	75.89	55.11	70.75	60.41	64.64	69.45
Mean ± SD (%)	80.69 ± 4.13					72.64 ± 3.79					64.67 ± 5.16				
SOSC	57.20	72.47	65.30	66.70	69.01	41.91	57.68	44.09	51.62	54.58	38.15	57.28	42.06	45.39	50.72
Mean ± SD (%)	66.57 ± 4.21					49.90 ± 5.65					46.52 ± 5.75				
APA	34.48	50.94	39.03	42.23	47.63	21.05	36.29	21.81	25.04	30.63	15.72	42.76	19.24	27.59	36.64
Mean ± SD (%)	26.48 ± 5.00					26.47 ± 5.32					28.01 ± 11.47				

Abbreviations: APA, air powder abrasion; CUR, curette; SD, standard deviation; SOSC, soundscaler.

**TABLE 4:** Medians, means, and standard deviations (%) of residual colored surface areas after (a) nonsurgical and (b) surgical treatment with 3.0 mm peri‐implant soft tissue thickness with three different decontamination methods (CUR, SOSC, APA). Separately presentation for different treatment methods and defect angulations. *N* = 10 for each treatment method in each defect angulation.

Defect angulation	30°					60°					90°				
			Percentile (%)					Percentile (%)					Percentile (%)		
	Min. (%)	Max. (%)	25th	50th (Median)	75th	Min. (%)	Max. (%)	25th	50th (Median)	75th	Min. (%)	Max. (%)	25th	50th (Median)	75th
(a) *Nonsurgical*															
CUR	44.59	86.28	74.22	78.15	80.46	62.46	78.63	73.08	75.32	77.48	35.05	84.00	70.53	73.23	81.15
Mean ± SD (%)	75.34 ± 11.47					74.40 ± 4.58					71.59 ± 13.87				
SOSC	61.47	85.53	69.54	73.15	79.79	49.37	67.84	57.91	64.18	66.85	44.02	59.50	46.73	54.33	56.85
Mean ± SD (%)	73.87 ± 7.66					61.97 ± 6.40					52.35 ± 5.65				
APA	83.31	97.10	92.96	95.16	96.71	74.60	97.64	86.17	90.81	91.91	63.82	89.69	82.54	84.48	87.42
Mean ± SD (%)	93.94 ± 4.15					88.81 ± 6.17					83.00 ± 7.16				
(b) *Surgical*															
CUR	69.98	83.70	72.37	73.93	78.20	65.43	78.05	67.38	70.53	75.71	65.34	75.91	68.09	71.60	74.18
Mean ± SD (%)	75.36 ± 4.33					71.20 ± 4.51					71.27 ± 3.62				
SOSC	52.52	76.99	55.14	60.25	68.70	47.13	54.34	47.90	50.75	52.26	28.05	46.43	36.17	39.81	44.74
Mean ± SD (%)	61.85 ± 7.85					50.53 ± 2.34					39.88 ± 5.74				
APA	36.44	62.82	38.38	43.15	49.30	14.96	52.29	18.35	25.74	30.62	10.91	49.50	14.82	17.76	23.59
Mean ± SD (%)	44.97 ± 8.45					27.09 ± 10.77					21.26 ± 10.94				

Abbreviations: APA, air powder abrasion; CUR, curette; SD, standard deviation; SOSC, soundscaler.

In detail, the use of APA in the surgical approach with a defect angle of 90° and a STT of 3.0 mm showed the greatest cleaning efficacy (Table 4b), while the use of APA in the nonsurgical approach with a defect angle of 60° and a STT of 1.5 mm showed the lowest cleaning efficacy (Table 3a).

### 3.3 Cleaning Method

Overall APA achieved the highest cleaning efficacy, followed by SOSC, while CUR showed the lowest efficacy (APA: 70.63%, SOSC: 69.62%, CUR: 81.48%; *p* < 0.05; *n* = 120). The major difference in cleaning efficacy of the decontamination method was found in the comparison of APA with CUR and SOSC with CUR, while the difference when comparing the method APA with SOSC was minor.

### 3.4 Approach

The ST approach demonstrated a significant better cleaning efficacy compared to the NST approach, when considered independently of the method, STT, and defect angle (ST: 82.65%, NST: 65.17%; *p* < 0.001; *n* = 180).

Each pairwise comparison of the methods within the approaches, irrespective of the STT and defect angle, revealed significant different cleaning efficacies. While APA in the ST approach showed the best performance followed by SOSC and CUR (APA > SOSC > CUR), in the NST approach SOSC was superior to CUR and APA (SOSC > CUR > APA) (NST: APA: 91.34%, SOSC: 73.87%, CUR: 82.73%; ST: SOSC: 65.38%, CUR: 80.22%, APA: 49.92%; *p* ≤ 0.001; *n* = 60). The cleaning efficacy within the ST approach was superior to that of the NST approach across each defect angle, when comparing independently of the method and STT (*p* < 0.001; *n* = 60).

### 3.5 Defect Angle

In comparison of the defect angles (30°, 60°, and 90°) independent of the method, approach and STT the cleaning efficacy at a 90° as well as at a 60° defect were significantly higher (90°: *p* > 0.001, 60°: *p* = 0.024; *n* = 120) than at 30° (30°: 79.04%, 60°: 73.40%, 90°: 69.29%).

As the defect angle increases, the cleaning efficacy of each method investigated increases independently of the approach and STT. However, these differences are only significant for SOSC (*p* < 0.001, *n* = 40). Only the comparison between SOSC and CUR within all three defect angles is significantly in favor of the cleaning efficacy with SOSC (*p* < 0.05, *n* = 40). While APA achieves the best cleaning efficacy at 30° (74.27%), APA (69.79%), and SOSC (69.12%) are similarly effective at 60°. SOSC achieves the best cleaning efficacy at 90° (60.83%).

### 3.6 SEM Analysis

Visual analysis of the SEM images showed surface damage on the implants treated with CUR or SOSC. Severe damage, in the form of scratches, was visible in implants cleaned with CUR and SOSC, treated with the ST approach and had a 1.5 mm STT according suprastructure. The SEM images of implants cleaned with APA that were treated with the ST approach showed the most similarities to the reference SEM image. In general, less surface damage was detected after the use of APA. No significant differences were observed in SEM images when comparing decontamination methods between the 1.5 and 3.0 mm STTs.

## 4 Discussion

This study aimed to analyze the in vitro efficacy of nonsurgical (NST) and surgical (ST) implant surface decontamination methods dependent on three different defect configurations, suprastructure geometries and different vertical peri‐implant soft tissue thicknesses (STT) of 1.5 or 3.0 mm. The overall cleaning efficacy decreased significantly (APA > SOSC > CUR). Analysis of variance revealed significant associations of color remnants with the approach, the method used, and the defect angulation (*p* < 0.001). The study failed to identify significant (*p* < 0.001) differences in the cleaning efficacy associated with the different STTs. SEMs showed less surface damages after use of APA.

To the best of the authors' knowledge, no comparable in vitro study exists on implant surface decontamination in relation to different STTs in the presence of correspondingly adapted suprastructures. Confirming previous studies with similar design, none of the examined implant surfaces were 100% free of color remnants (Francis et al. 2022; Iatrou et al. 2021; Keim et al. 2019; Korello et al. 2023; Ronay et al. 2017; Sahrmann et al. 2015; Tuchscheerer et al. 2021).

### 4.1 Soft Tissue Thickness

Analysis of variance failed to show significant associations between color remnants and the STT. The present data were more inhomogeneous than the data of similar in vitro studies, as STT was introduced as additional factor (Iatrou et al. 2021; Korello et al. 2023; Ronay et al. 2017). This led to reduced group sizes compared with previous mentioned studies, which could be a possible reason why no differences could be found between 1.5 and 3.0 mm STT. In contrast to almost comparable studies (Atieh et al. 2023; Chan et al. 2019; Katafuchi et al. 2018; Mattheos et al. 2021; Puisys et al. 2023; Wang et al. 2022; Yi et al. 2020; Zhang et al. 2020), the focus of the present study is not on the description of the implant‐prosthetic interface in terms of a protective design, but on whether the dimension of the interface, that is, the combination of emergence angle and STT, may have an influence on the cleaning efficacy in the event of peri‐implantitis. Regarding the outcome of this study, cleaning efficacy might allow some flexibility in the design of the emergence profile and the STT. However, aesthetics as well as prevention of peri‐implant diseases are limitations, which must be given greater consideration.

### 4.2 Cleaning Method

When considering the decontamination method irrespective of the approach, defect angle, and STT, APA showed the best cleaning efficacy, followed by SOSC and CUR this verifies the superiority of APA stated in several in vitro studies (Iatrou et al. 2021; Keim et al. 2019; Korello et al. 2023; Ronay et al. 2017; Sahrmann et al. 2015). A systematic review on titanium surface decontamination in In Vitro studies further confirmed this (Francis et al. 2022). Another in vitro study examined the use of hydroxyapatite‐coated titanium implant surfaces in ST approaches without the use of suprastructures, reporting that SOSC demonstrated superior cleaning efficacy compared to CUR and APA (Munakata et al. 2022). The superiority of SOSC to APA mentioned in the study of Munakata et al. (2022) might be explained by the lack of powder reflection in a class Ie defect (Schwarz et al. 2007), as well as the use of different powder of the APA device (ß‐tricalcium phosphate powder).

### 4.3 Approach

Considering the approach independently cleaning efficacy was significantly higher in the ST approach, than in the NST approach. In the present study, the NST data for CUR and SOSC showed comparable results to those of Ronay et al. (2017), which were observed without a suprastructure and with a consistent STT of 3.0 mm. Compared with the study of Iatrou et al. (2021), who used a mucosa mask simulating a STT of 3.0 mm without a suprastructure, the present study revealed higher color remnant values for each method. This difference may have been caused by inferior accessibility due to neighboring teeth and the presence of a suprastructure in the current study, as the type of implants used between the studies were similar. Compared with Korello et al. (2023), who used a suprastructure and a mucosa mask with a consistent STT of 3.0 mm, lower color residues for CUR and SOSC were observed in the current study. This could be caused by the fact of different experienced examiners. However, APA failed to show superior cleaning performance compared to SOSC and CUR within the NST approach. In the study by Korello et al. (2023), neighboring teeth, a suprastructure, and a silicone mucosa mask were implemented. Nonetheless, the emergence profile of the suprastructure was not explained in significant detail. Therefore, an assumption can be made that due to the presence of a suprastructure and mucosa mask in the NST approach, the accessibility by the nozzle tip might have been limiting in the present study caused by the adhesion of silicone to the implant surface. This might be a specific problem of the method of this In Vitro study, as it differs to the rigidity of inflamed peri‐implant tissues.

### 4.4 Defect Angle

Several studies have examined the relationship between the defect angle and cleaning efficacy. In the present study, a higher cleaning efficacy is observed at greater defect angles when comparing defect angles irrespective of further factors. While APA shows better results in narrow defects (30°), SOSC is superior in wider defects (90°). With regard to the defect geometry, effects such as the reflection of powder particles (APA) and the formation of microcurrents (SOSC) could play a major role. Iatrou et al. (2021), who used a mucosa mask simulating a STT of 3.0 mm without any suprastructure, stated that CUR, APA and SOSC performed best with a defect angulation of 60° and worst with 30°. Keim et al. (2019) examined CUR, APA and SOSC in a surgical approach, while CUR performed best in terms of cleaning efficacy with a defect angle of 90°, APA and SOSC performed best with a defect angle of 60°. A better cleaning efficacy at higher defect angles is ascertained for APA in a ST approach (Sahrmann et al. 2015, 2013), for APA and SOSC in a NST approach (Ronay et al. 2017) and for APA, SOSC and CUR in ST and NST approaches (Korello et al. 2023). This evidence is further expanded by another In Vitro study that verified the In Vitro cleaning efficacy of APA and titanium brushes in ST approaches with better performance in defect Classes Ib/Ic (Schwarz et al. 2007), that allow easy access from the buccal side, performed without suprastructures or mucosa masks (Luengo et al. 2022).

### 4.5 SEM Analyses

An In Vitro study from 2021 reported visible damage in SEM images following CUR, and, to a lesser extent, SOSC, as well as poorer debridement success following CUR (Secgin‐Atar et al. 2021). That In Vitro study used natural oral biofilms by using explanted implants and an ST approach without using suprastructures. Further studies confirm the gentle debridement of APA observed in this In Vitro study regarding surface damage to the implant (Iatrou et al. 2021; Keim et al. 2019; Korello et al. 2023; Ronay et al. 2017; Tuchscheerer et al. 2021).

This study has several limitations, as it simulated a clinical setting using an In Vitro model. Color was used to imitate an oral biofilm, which might have a different adhesion strength compared to natural biofilms; however, it enabled easy detection and comparison to former In Vitro studies (Iatrou et al. 2021; Keim et al. 2019; Korello et al. 2023; Ronay et al. 2017; Sahrmann et al. 2015; Tuchscheerer et al. 2021). The rigidity of the mucosa mask, adhesion of the silicone to the implant surface differed from the peri‐implant soft tissues of the human oral cavity and access might be more limited through the tongue, cheek, and stoma (Korello et al. 2023). Moreover, the tissue turgor was much higher through the greater horizontal volume of the mucosa mask compared with the diameter of peri‐implant soft tissue. Given to the study design with 10 implants per group, the nonsignificant result might be a statistical power issue. The analysis is another limiting factor. The photographs were taken from two sides (180°) and not from further angles (Iatrou et al. 2021; Keim et al. 2019; Korello et al. 2023; Tuchscheerer et al. 2021), so that parts of the surface remained disregarded and consistent light reflection occurred. Nevertheless, an In Vitro approach enables a comparison of different methods and therefore seems to be best suitable to evaluate and test the given hypothesis. To confirm the presence of a correlation between cleaning efficacy and emergence angle, as well as vertical peri‐implant soft tissue thickness, further studies are required, that include human clinical trials, In Vitro studies that investigate different accurate numeric emergence angle or differentiation in the three zones of the emergence profile of the suprastructure (Gomez‐Meda et al. 2021), the use of bacterial biofilm as plaque, larger study groups to increase the statistical power, as well as precise three‐dimensional surface detection of color remnants and analysis.

## 5 Conclusions

This In Vitro study failed to show significant influence of STT and corresponding adaptation in the design of the suprastructure to the efficacy of implant surface decontamination. However, used method, defect angle and the approach were confirmed as influencing factors for the cleaning efficacy.

## Author Contributions

**Annika Rahner:** data curation, writing – original draft, investigation, writing – review and editing. **Peter Eickholz:** methodology, writing – review and editing, conceptualization. **Jan‐Frederik Gueth:** conceptualization, methodology, writing – review and editing. **Tuǧba Zahn:** conceptualization, writing – review and editing, investigation. **Iulia Dahmer:** writing – review and editing, formal analysis. **Hari Petsos:** conceptualization, writing – original draft, funding acquisition, writing – review and editing, formal analysis, supervision, project administration.

## Ethics Statement

This article does not contain any studies with human participants or animals performed by any of the authors. An exemption letter from the Institutional Review Board for Human Studies of the Medical Faculty of the Goethe University Frankfurt/Main has been provided approving that no ethical concerns are raised.

## Conflicts of Interest

The authors declare no conflicts of interest.

## Supporting information


**Figure S1.** Schematic drawing of a lower STT (a) with a correspondingly flatter emergence angle and a higher STT with a correspondingly steeper emergence angle with an identical emergence profile in the area exiting the peri‐implant mucosa in both cases.

## Data Availability

The data that support the findings of this study are available from the corresponding author upon reasonable request.
