# Supplementary figures and images for: In Vitro Assessment of the Potential Effect of Vertical Peri‐Implant Soft Tissue Thickness on Nonsurgical and Surgical Implant Surface Decontamination Methods
Source: Clin Oral Implants Res. 2025 Feb 10;36(6):683–97. doi: 10.1111/clr.14415 (PMC12146503; doi:10.1111/clr.14415)

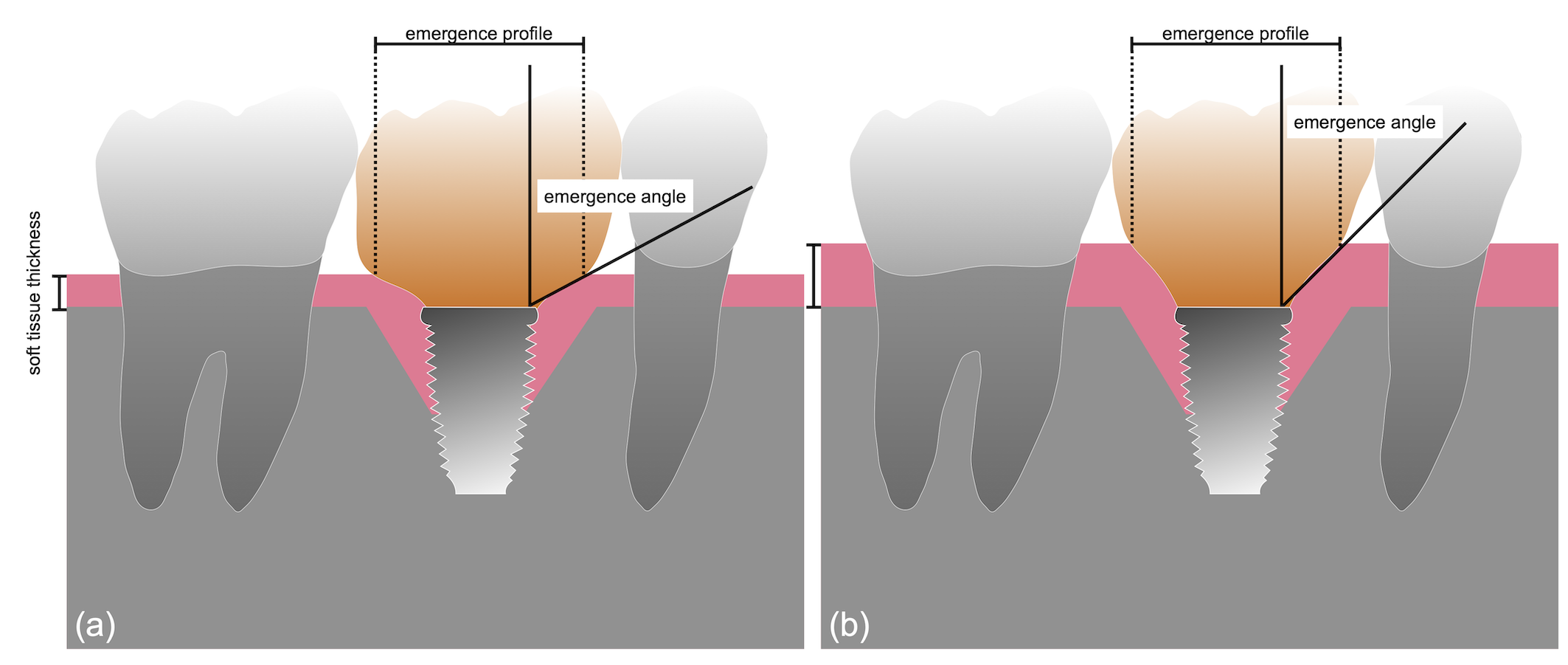

Supplement: Supplementary file 1 — Figure S1. Schematic drawing of a lower STT (a) with a correspondingly flatter emergence angle and a higher STT with a correspondingly steeper emergence angle with an identical emergence profile in the area exiting the peri‐implant mucosa in both cases. [file CLR-36-683-s001.tiff]
